# Plasma trimethylamine-N-oxide, its precursors and risk of cardiovascular events in patients with acute coronary syndrome: Mediating effects of renal function

**DOI:** 10.3389/fcvm.2022.1000815

**Published:** 2022-09-21

**Authors:** Raul Sanchez-Gimenez, Óscar M. Peiró, Gil Bonet, Anna Carrasquer, Georgios A. Fragkiadakis, Mònica Bulló, Christopher Papandreou, Alfredo Bardaji

**Affiliations:** ^1^Department of Cardiology, Joan XXIII University Hospital, Tarragona, Spain; ^2^Institut d'Investigació Sanitària Pere Virgili (IISPV), Reus, Spain; ^3^Department of Medicine and Surgery, Rovira i Virgili University, Tarragona, Spain; ^4^Department of Nutrition and Dietetics Sciences, School of Health Sciences, Hellenic Mediterranean University (HMU), Siteia, Greece; ^5^Department of Biochemistry and Biotechnology, Rovira i Virgili University, Reus, Spain; ^6^Centro de Investigación Biomédica en Red Fisiopatología de la Obesidad y la Nutrición (CIBEROBN), Instituto de Salud Carlos III, Madrid, Spain

**Keywords:** trimethylamine-N-oxide, metabolites, acute coronary syndrome, major adverse cardiovascular events, prognostic

## Abstract

**Aims:**

To examine associations of the gut microbial metabolite trimethylamine-N-oxide (TMAO) and its precursors with risk of cardiovascular events in acute coronary syndrome (ACS), and determine whether these associations were mediated by renal function.

**Methods:**

In this prospective cohort study, we included 309 patients with ACS. During a mean follow-up of 6.7 years, 131 patients developed major adverse cardiovascular events (MACE) (myocardial infarction, hospitalization for heart failure, and all-cause mortality). Plasma concentrations of TMAO, trimethylamine (TMA), choline, betaine, dimethylglycine and L-carnitine were profiled by liquid chromatography tandem mass spectrometry. Hazard ratios were estimated with multivariable Cox regression models. The mediating role of estimated glomerular filtration rate (eGFR) was tested under a counterfactual framework.

**Results:**

After adjustment for traditional cardiovascular risk factors and medications, participants in the highest tertile vs. the lowest tertile of baseline TMAO and dimethylglycine concentrations had a higher risk of MACE [(HR: 1.83; 95% CI: 1.08, 3.09) and (HR: 2.26; 95% CI: 1.17, 3.99), respectively]. However, with regards to TMAO these associations were no longer significant, whereas for dimethylglycine, the associations were attenuated after additional adjustment for eGFR. eGFR mediated the associations of TMAO (58%) and dimethylglycine (32%) with MACE incidence. The associations between dimethylglycine and incident MACE were confirmed in an internal validation. No significant associations were found for TMA, choline, betaine and L-carnitine.

**Conclusion:**

These findings suggest that renal function may be a key mediator in the association of plasma TMAO with the development of cardiovascular events after ACS. The present findings also support a role of dimethylglycine in the pathogenesis of MACE, which may be mediated, at least partially, by renal function.

## Introduction

Acute coronary syndrome (ACS) is a leading cause of morbidity, mortality, and health care cost worldwide (1, 2). Patients with ACS are at high risk for recurrent cardiovascular events (3, 4) and therefore secondary prevention is paramount. Although many prognostic tools have been developed for, efforts are still ongoing to identify reliable and predictive biomarkers, which may help predict clinical outcomes of high-risk ACS patients (5). Given the fact that metabolic perturbations are frequent in ACS (6, 7), metabolomics seems a promising approach to reveal these perturbations and to identify metabolite-based biomarkers to aid in both the identification of pathophysiological processes relevant to adverse events development and improve preventive clinical outcomes reduction efforts after ACS.

Lately, the gut microbiota-related metabolite trimethylamine-N-oxide (TMAO) has attracted considerable attention across cardiovascular research due to its association with the risk of cardiovascular outcomes (8). Since the discovery of a relationship between TMAO and major adverse cardiovascular events (MACE) in ACS patients, in 2017 (9), few studies were conducted and a recent meta-analysis of prospective studies revealed positive pooled estimates with substantial methodologic heterogeneity among the studies indicating the need for more prospective researches to evaluate this relationship (10). Little evidence also exists on the relationship between TMAO precursors and risk of MACE after ACS. A previous study suggested that higher choline and betaine concentrations in plasma were associated with greater risk of incident MACE, but only among those ACS patients with concomitantly elevated plasma TMAO concentrations (11). In another small study with short follow-up time, plasma dimethylglycine, the immediate product of betaine, was associated with increased risk of all-cause death, acute myocardial infarction (MI), and hospitalization for heart failure (12); still, associations with a composite of these clinical events have not been examined. In addition, the prognostic role of trimethylamine (TMA), the immediate precursor of TMAO, and L-carnitine in ACS remains unknown. Because circulating levels of these metabolites have been inversely correlated with levels of the renal function marker, glomerular filtration rate (GFR), in previous studies (13–16) and some studies suggest that GFR can be a mediator in these associations (17, 18) further studies exploring the mediating role of renal function are needed.

The aim of the present prospective cohort study was (i) to investigate associations between baseline plasma concentrations of TMAO and its precursors with incident MACE among patients with ACS and (ii) to determine whether these associations are mediated by renal function.

## Materials and methods

### Study design and participants

The study was approved by the Institutional Ethical Committee and was conducted in accordance with the ethical principles set forth of the Declaration of Helsinki. All patients gave their written consent for participation in the study.

Patients with ACS admitted to Joan XXIII University Hospital and underwent a coronary angiography from January 2011 to May 2013, were included in this study and followed-up until 2022. The European Society of Cardiology guidelines were followed to define ACS as follows: patients with ST-segment elevation myocardial infarction (STEMI), non-ST-segment elevation myocardial infarction (NSTEMI) and unstable angina. Acute myocardial infarction (STEMI or NSTEMI) was diagnosed according to the current universal definition of myocardial infarction (19). Unstable angina was defined as the presence of ACS symptoms at rest or with minimal exertion in the absence of cardiomyocyte necrosis (20). Patients that suffered a MI other than type 1, and those with foreign residency were excluded from this study. The Joan XXIII University Hospital is a tertiary hospital located in South Europe (Tarragona, Spain) and the only hospital in the region for performing angiograms and percutaneous coronary interventions. This could result in difficulties to follow-up patients treated at the hospital during the acute phase. Therefore, many patients were only treated at the hospital during the acute phase and then followed-up by their local cardiologist after discharge.

### Ascertainment of MACE cases

Information on clinical outcomes was obtained by contacting patients every year and by analysis of the hospital's patient management information system [myocardial infarction (ICD-10-CM I21), hospitalization for heart failure (ICD-10-CM I50)]. MACE was defined as the composite of myocardial infarction, hospitalization for heart failure, and all-cause mortality.

### Metabolomic profiling

Blood samples were collected into EDTA tubes from patients during coronary angiography. These samples were shipped from the Joan XXIII University Hospital to the Biobank of the Pere Virgili Health Research Institute, registered in the National Biobank Network, for further processing and storage. After centrifugation, plasma aliquots were stored at −80°C until analysis. TMAO, TMA, choline, betaine, dimethylglycine and L-carnitine were quantified by stable-isotope dilution method using liquid chromatography coupled to tandem mass spectrometry. Samples (50 μl plasma) were mixed with 10 μl of internal standard, 75 μl of 50 mM tert-Butyl bromoacetate in ACN and 10 μl of 70% ammonium hydroxide. Samples were vortexed for 1 min and incubated for 30 min at room temperature. A volume of 50 μl of 1% formic acid in ACN was added. Samples were centrifuged for 5 minat 15,000 rpm and 4°C. Samples were chromatographed on an ACQUITY UPLC BEH HILIC, 1.7 μm 2.1 × 100 mm (Waters, Milford, MA, USA) and in junction with a UHPLC 1290 Infinity II (Agilent Technologies, Santa Clara, CA, USA). The gradient consisted of a gradient of 0% A for 1 min, to 10% A at 4 min, to 55% A at 5 min and 55% A for 2 min. Mobile phase A was composed of 10% acetonitrile and 90% water with 10 mmol/L of ammonium formate and 0.125% formic acid; mobile phase B was 90% acetonitrile and 10% water with 10 mmol/L of ammonium formate and 0.125% formic acid. Flow rate was kept constant at 0.5 mL/min, and the column manager was set at room temperature for the duration of the sequence. Agilent QqQ/MS 6470 Series with an electrospray ionization probe operating in positive-ion mode was used for mass spectrometric analysis. The source conditions were set at 50 psi for the nebulizer gas, 300°C for the gas temperature, 11 L/min for the gas flow, 400°C for the sheath gas temperature, 12 L/min for the sheath gas flow, 2,500 V for the capillary voltage, and 500 V for the nozzle voltage. Quantitative determination was performed using the multiple reaction monitoring mode, and the transitions for each compound are detailed in Supplementary Table S1. Assay quality assurance was monitored by routine analysis of pooled quality control plasma. Information about the mass to charge ratio and retention time is shown in Supplementary Table S1. The metabolite identification confidence level according to the published criteria (21) is Level 1: confirmed structure with MS, MS^2^, RT and reference standard.

### Covariates

Information about demographics, smoking status, medical conditions, and medication use were collected during hospital admission. Body mass index (BMI) was calculated as weight divided by height squared (kg/m^2^). The estimated GFR (eGFR) was calculated by using the Chronic Kidney Disease Epidemiology Collaboration creatinine equation (22). Participants were considered having type 2 diabetes (T2D), dyslipidemia, or hypertension if they had previously been diagnosed.

### Statistical analyses

Baseline characteristics of patients are described as mean ± standard deviation (SD) or median (interquartile range) for quantitative variables and percentages for categorical variables. A natural logarithmic transformation in metabolites' concentrations was applied to approximate a normal distribution.

We used crude and 2 multivariable Cox proportional hazards models for time-to-event analysis to determine hazard ratios (HRs) and 95% confidence intervals (CIs) for MACE. We checked the proportional hazards assumption by examining the Schoenfeld residuals and found that the assumption was not violated. The first multivariable-adjusted Cox regression model (multivariable model 1) was fitted as follows: multivariable model 1 was adjusted for age, sex, BMI (kg/m^2^), smoking (never, current, or former), hypertension (yes or no), dyslipidemia (yes or no), T2D (yes or no), unstable angina (yes or no), acute ST-segment elevation myocardial infarction (yes or no), non-ST-segment elevation acute myocardial infarction (yes or no), statin medication (yes or no), beta-blockers (yes or no), oral antidiabetic medication (yes or no), insulin medication (yes or no), diuretics (yes or no), and aspirin (yes or no). The multivariable model 1 was additionally adjusted for eGFR (multivariable model 2) to further assess the effect of renal function. Spearman's correlation analyses were performed to investigate whether there was a relationship between circulating levels of TMAO, TMA, choline, betaine, L-carnitine and eGFR.

Metabolites were analyzed as both continuous variables [1-standard deviation (SD) (1-SD) increment in their ln-transformed levels] and by using tertiles. To appraise the linear trend across tertiles, the median metabolite concentration within each tertile was included in the Cox regression models as a continuous variable. Bootstrap resampling of the original sets (1,000-fold) was performed to internally validate the associations. Non-parametric associations were examined by fitting cubic splines to the Cox regression model. Tests for non-linearity involved the likelihood ratio test, comparing the model with only the linear term to the model with the linear and the cubic spline terms. These analyses were performed using Stata 14.1 (Stata Corp.), at a two-tailed α of 0.05.

We carried out a counterfactual-based mediation analysis (23) to explore the possibility that the association of plasma metabolites concentrations with MACE incidence might be mediated by eGFR. Mediation analysis under the counterfactual framework has advantages over conventional mediation analysis since it enables the incorporation of exposure-mediator interactions, adjusts for measured intermediate confounders, and deals with non-linear relationships. By performing this analysis using the R package “medflex” (24), the total effect of metabolites on MACE risk can be decomposed into a natural direct effect of metabolites on MACE risk and a natural indirect effect of metabolites accounted by the effect of the putative mediators, in our case the eGFR. We also accounted for potential confounding by the covariates included in the multivariable Cox regression model 1. The bootstrapping technique with 1,000 replications was employed to derive the 95% confidence intervals. The mediation analysis was performed using Poisson based Generalized Linear Models by splitting the follow-up time every time an event was observed [survSplit() function in the survival package] in such a way every time interval contains only one event and thereafter we estimated the effects of the model parameters (25, 26) as Cox regression models are currently not implemented in “medflex”. It has been suggested that a similar hazard ratio to that of the Cox regression model could be obtained by fitting a Poisson model on survival data (27, 28). To approximate more the Cox results we added a time term to the Poisson model. The mediated proportion was calculated as natural indirect log-incident rate ratios (IRR) divided by total effect log-IRR. This analysis was performed using R version 4.2.0 (R Foundation for Statistical Computing, Vienna, Austria).

## Results

### Baseline characteristics of patients

Baseline characteristics of this cohort are shown in Table 1. A total of 309 patients with ACS were included in the study. The mean age was 64.9 years and 28.8% were women. The mean values of BMI and eGFR were 28.1 kg/m^2^ and 81.3 mL/min/1.73 m^2^, respectively. Of all patients, 62.0% were admitted with NSTEMI, 22.0% with STEMI and 15.9% with unstable angina. Half of them were following statins medication, followed by those taking aspirin (40.5%), beta-blockers (30.4%), diuretics (25.2%) and oral antidiabetic agents (22.7%). The median (interquartile range) of metabolites' concentrations can also be seen in Table 1.

**Table 1:** Baseline characteristics of patients.

**Characteristics**	**Patients (*N* = 309)**
Age (years)	64.9 ± 12.3
**Sex, %**
Women	28.8
Men	71.2
Body mass index, kg/m^2^	28.1 ± 4.0
eGFR (mL/min/1.73 m^2^)	81.3 (62.1–96.7)
Type 2 diabetes, %	37.2
Hypertension, %	67.6
Dyslipidemia, %	60.8
**Smoking, %**
Never	35.6
Former	33.9
Current	30.4
**Discharge diagnostic, %**
Unstable angina	15.9
STEMI	22.0
NSTEMI	62.0
**Medications, %**
Statins	50.5
Beta-blockers	30.4
Aspirin	40.5
Diuretics	25.2
Oral antidiabetic agents	22.7
Insulin medication	8.7
**Metabolites**
TMAO, μmol/L	9.98 (7.42–19.19)
TMA, nmol/L	49.41 (32.17–88.32)
Choline, μmol/L	11.81 (9.59–14.30)
Betaine, μmol/L	41.01 (32.46–50.62)
Dimethylglycine, μmol/L	3.26 (2.42–4.19)
L-carnitine, μmol/L	47.26 (40.0–57.0)

Continuous data are presented as mean ± standard deviation or median (interquartile range), and categorical variables are presented as %. eGFR, estimated glomerular filtration rate; STEMI, ST elevation myocardial infarction; NSTEMI, non-ST elevation myocardial infarction, TMAO, trimethylamine N-oxide; TMA, trimethylamine.

### Baseline metabolites and risk of MACE

ACS patients were followed for a mean of 6.7 (SD = 3.6) years for MACE occurrence. The associations of TMAO and its precursors with MACE risk are displayed in Table 2. In the multivariable model 1, the estimated HR for incident MACE reached significance only in the highest, compared with the lowest tertile of plasma concentrations of TMAO (HR: 1.83; 95% CI: 1.08, 3.09). Furthermore, the risk of MACE significantly increased per 1-SD increase in TMAO concentrations (HR: 1.25; 95% CI: 1.07, 1.46; *p* = 0.005). Spearman's correlation analyses revealed a significant negative correlation between plasma TMAO and eGFR levels (r = −0.40, *P* < 0.001) (Supplementary Figure S1A). Importantly, the associations between TMAO and risk of MACE were no longer significant after additional adjustment for eGFR. With regard to dimethylglycine, in the multivariable model 1 the estimated HR for incident MACE in the highest compared with the lowest tertile was 2.56 (95% CI: 1.44, 4.54; *p* trend = 0.001) and this association remained significant after inclusion of eGFR into the Cox regression model (HR: 2.26; 95% CI: 1.17, 3.99; *p* trend = 0.012). Kaplan–Meier survival analysis demonstrated a significant difference in MACE incidence across tertiles of dimethylglycine concentration subgroups (log-rank, *P* < 0.001; Supplementary Figure S2). Similarly, significant positive associations with MACE incidence were observed for dimethylglycine when was modeled continuously (per 1 SD) in the multivariable model 1 (HR: 1.53; 95% CI: 1.23, 1.90; *p* < 0.001) and these associations were attenuated after adjusting for eGFR but remained statistically significant (HR: 1.41; 95% CI: 1.10, 1.81; *p* = 0.007). Dimethylglycine concentrations were inversely correlated with eGFR levels (r = −0.29, *P* < 0.001) (Supplementary Figure S1E). Cubic spline curves (Supplementary Figure S3) showed that dimethylglycine was non-linearly associated with MACE risk (*P*-value for non-linearity with MACE risk was = 0.003). Internal validation by bootstrap resampling confirmed the significant findings for dimethylglycine (Supplementary Table S3). No significant associations for TMA, choline, betaine, L-carnitine and MACE risk were observed (Table 2).

**Table 2:** Associations of baseline individual metabolites concentrations with the risk of MACE^a^.

		**Tertiles of plasma metabolite concentrations**				
**Metabolite**	**T1**	**T2**	**T3**	***P* trend**	**HR per 1 SD increment**	***P*-value**
**TMAO**
Concentrations (μmol/L)	<8.01	8.01– <14.96	>14.96			
Cases	29	45	57			
Crude model	Ref.	1.81 (1.15–2.85)	2.63 (1.69–4.08)	**<0.001**	1.42 (1.24–1.64)	**<0.001**
MV1	Ref.	1.45 (0.85–2.46)	1.83 (1.08–3.09)	**0.043**	1.25 (1.07–1.46)	**0.005**
MV2	Ref.	1.43 (0.84–2.42)	1.66 (0.98–2.82)	0.119	1.15 (0.96–1.37)	0.121
**TMA**
Concentrations (nmol/L)	<36.09	36.09– <70.24	>70.24			
Cases	38	45	48			
Crude model	Ref.	1.33 (0.87–2.04)	1.47 (0.95–2.25)	0.117	1.11 (0.95–1.29)	0.187
MV1	Ref.	1.18 (0.73–1.92)	1.21 (0.75–1.96)	0.534	1.05 (0.89–1.25)	0.568
MV2	Ref.	1.13 (0.70–1.83)	1.10 (0.68–1.81)	0.792	1.01 (0.85–1.21)	0.894
**Choline**
Concentrations (μmol/L)	<10.39	10.39– <13.35	>13.35			
Cases	38	42	51			
Crude model	Ref.	1.14 (0.74–1.75)	1.66 (1.08–2.55)	**0.019**	1.37 (1.16–1.61)	**<0.001**
MV1	Ref.	0.99 (0.61–1.63)	0.96 (0.58–1.58)	0.865	1.15 (0.95–1.40)	0.146
MV2	Ref.	0.99 (0.61–1.63)	0.87 (0.53–1.40)	0.530	1.02 (0.84–1.24)	0.840
**Betaine**
Concentrations (μmol/L)	<35.27	35.27– <47.02	>47.02			
Cases	48	38	45			
Crude model	Ref.	0.75 (0.50–1.14)	0.99 (0.66–1.48)	0.964	1.02 (0.84–1.23)	0.863
MV1	Ref.	0.70 (0.43–1.12)	0.84 (0.50–1.40)	0.586	0.97 (0.77–1.22)	0.771
MV2	Ref.	0.64 (0.39–1.03)	0.80 (0.49–1.31)	0.504	0.96 (0.77–1.20)	0.742
**Dimethylglycine**
Concentrations (μmol/L)	<2.69	2.69– <3.80	>3.80			
Cases	31	46	54			
Crude model	Ref.	1.60 (1.02–2.50)	2.31 (1.49–3.60)	**<0.001**	1.47 (1.22–1.76)	**<0.001**
MV1	Ref.	1.63 (0.94–2.84)	2.56 (1.44–4.54)	**0.001**	1.53 (1.23–1.90)	**<0.001**
MV2	Ref.	1.53 (0.89–2.65)	2.16 (1.17–3.99)	**0.012**	1.41 (1.10–1.81)	**0.007**
**L-carnitine**
Concentrations (μmol/L)	<42.51	42.51– <53.74	>53.74			
Cases	43	39	49			
Crude model	Ref.	0.88 (0.57–1.36)	1.20 (0.80–1.81)	0.316	1.01 (0.82–1.24)	0.934
MV1	Ref.	0.71 (0.43–1.16)	1.23 (0.74–2.03)	0.281	1.06 (0.85–1.32)	0.622
MV2	Ref.	0.77 (0.47–1.26)	1.21 (0.73–2.00)	0.352	1.06 (0.87–1.30)	0.548

^a^Values are HR (hazard ratios) with 95% confidence intervals. A natural logarithmic transformation was applied to the raw values of individual metabolites. Cox regression analysis was used. MV1 adjusted for age, sex, body mass index (kg/m^2^), smoking, hypertension, dyslipidemia, type 2 diabetes, unstable angina, acute ST-segment elevation myocardial infarction, non-ST-segment elevation acute myocardial infarction, statin medication, beta-blockers, oral antidiabetic medication, insulin medication, diuretics, aspirin. MV2 additionally adjusted for estimated glomerular filtration rate.

MV, multivariable; Ref, reference; MACE, major adverse cardiovascular events; TMAO, trimethylamine N-oxide; TMA, trimethylamine.

Bold text indicates statistically significant *P*-values.

### Mediation of the association between TMAO and dimethylglycine with MACE

Results from the mediation analyses are displayed in Table 3. Overall 58% of the total effect of TMAO (per 1-SD increase) on risk for MACE was mediated through eGFR, with natural direct effect of IRR: 1.15 (95% CI: 0.98–1.34) and natural indirect effect of IRR: 1.22 (95% CI: 1.10–1.34). Significant indirect effect on MACE risk was also found for dimethylglycine (per 1-SD increase). The magnitude of mediation was 32%, with natural direct effect of IRR: 1.17 (95% CI: 0.94–1.44) and natural indirect effect of IRR: 1.09 (95% CI: 1.01–1.19).

**Table 3:** Medflex counterfactual mediation analysis for MACE incident rate ratios (IRR) per 1 SD increment in TMAO and dimethylglycine concentrations; proportion mediated by eGFR with bootstrap 95% confidence intervals (1,000 iterations).

**Metabolite**	**Effect**	**IRR**	**95% lower CI**	**95% upper CI**	**Proportion mediated**
TMAO	Natural direct effect	1.15	0.98	1.34	58%
	Natural indirect effect	1.22	1.10	1.34	
	Total effect	1.38	1.22	1.55	
Dimethylglycine	Natural direct effect	1.17	0.94	1.44	32%
	Natural indirect effect	1.09	1.01	1.19	
	Total effect	1.28	1.04	1.54	

MACE, major adverse cardiovascular events; TMAO, trimethylamine N-oxide; eGFR, glomerular filtration rate; CI, confidence intervals.

## Discussion

This prospective cohort study of 309 patients with ACS demonstrated that baseline plasma concentrations of TMAO and dimethylglycine were associated with higher risk of MACE after a mean follow-up of 6.7 years. Notably, the strong positive associations for TMAO were attenuated and became insignificant after adjustment for eGFR. Importantly, eGFR, a marker of renal function, fully mediated the association between TMAO and MACE. Additionally, eGFR acted as a partial mediator in the relationship between dimethylglycine and MACE. No significant risk associations were observed for other TMAO precursors TMA, choline, betaine and L-carnitine.

### TMAO and MACE

To our knowledge, data on the relationship between TMAO and risk of MACE in patients with ACS are limited to prospective cohort studies from USA, UK, New Zealand, China and Japan that were recently meta-analyzed and revealed pooled HR estimates of 1.87 (95% CI: 1.41, 2.47) (10). Our study is the first conducted in ACS patients from the Mediterranean region with a long follow-up and high median TMAO concentrations of 9.98 μmol/L vs. 2.87–7.50 μmol/L in the studies included in the previous meta-analysis. Differences in genetic variation, dietary habits and gut microbiota composition may have affected the concentrations of TMAO in ACS patients from different geographical locations. Our results suggested that patients with elevated plasma TMAO concentrations were at high risk of developing MACE, after extensive adjustment for traditional cardiovascular risk factors and medications. However, the association of TMAO with MACE became insignificant after adjustment for eGFR, raising the possibility the renal function may mediate this association. Of note, we found a negative correlation between TMAO and eGFR, in agreement with previous studies among patients with chronic kidney disease (CKD) (13) and the general population (17), implying that TMAO may accumulate in the circulation in patients with impaired renal function. Therefore, TMAO could be a marker of reduced renal clearance. However, studies in mice suggest that TMAO may directly contribute to decreased renal function (29). Thus, it is possible that elevated circulating concentrations of TMAO may have a direct effect on renal impairment which in turn is a determinant of MACE (30). Our mediation analysis provides further evidence by documenting a strong mediating role of eGFR in the relationship between TMAO and MACE, and extend prior results on the mediating role of renal function in the association between TMAO and all-cause mortality (18). Understanding how renal function underlies the relation between TMAO and MACE risk is of great public health importance given the coexistence of reduced renal function in ACS patients (31).

### Dimethylglycine and MACE

Our findings concerning the positive relationship between dimethylglycine, a product of choline metabolic pathway, and MACE incidence in ACS patients extend the current knowledge of the role of this metabolite in cardiovascular health. Although unexplored in relation to a composite of cardiovascular events, higher concentrations of this metabolite were previously found to be associated with risk of acute myocardial infarction, and hospitalization for heart failure after ACS (12), incident acute myocardial infarction in patients with stable angina pectoris (32), atrial fibrillation and heart failure in individuals at high cardiovascular risk (33) and mortality in patients with coronary heart disease (34). In our study, the association between dimethylglycine and MACE risk was non-linear and more pronounced among patients with higher dimethylglycine concentrations.

Dimethylglycine, the by-product of the betaine-homocysteine methyltransferase (BHMT) reaction which catalyzes the transfer of methyl groups from betaine to homocysteine to form dimethylglycine and methionine (35), is oxidatively demethylated to sarcosine (36). Elevated concentrations of dimethylglycine could be caused either by a decreased activity of the enzyme dimethylglycine dehydrogenase that converts dimethylglycine to sarcosine or by inflammation and oxidative stress in ACS (37, 38) altering the flux through BHMT (39). It has been suggested that dimethylglycine may be related to adverse cardiovascular outcomes through its effect on the regulation of lipid and energy metabolism (32).

Adding eGFR to the Cox regression model attenuated the association between dimethylglycine and MACE risk. We also found an inverse association between plasma dimethylglycine concentrations and eGFR, in agreement with studies among patients with renal failure (40) and patients with stable angina pectoris (32). Notably, eGFR partially mediated this association offering new insight into understanding the relationship between dimethylglycine, renal function, and cardiovascular events. Given that declined renal function is a risk factor for MACE, we might speculate that there may be a synergistic relationship of increased dimethylglycine concentrations and decreased renal function with the increased risk for developing MACE.

CKD is defined for patients younger than 40 years by eGFR below 75 mL/min/1.73 m^2^; between 40 and 65 years by 60 mL/min/1.73 m^2^; for older than 65 years without albuminuria or proteinuria by eGFR below 45 mL/min/1.73 m^2^ (41). Since the eGFR range in our sample was close to normal renal function (age 64.9 ± 12.3, eGFR 62.1–96.7), further studies are necessary to quantitate the effects of TMAO and dimethylglycine on MACE during the development of CKD.

### Strengths and limitations

The main strengths of this study include its prospective design with a long-term follow-up. Furthermore, the extensive measure of metabolites involved in TMAO biosynthesis, may broaden our understanding of the metabolic processes related to TMAO pathway and the development of cardiovascular events in ACS.

With regard to limitations, even though we adjusted for many potential confounders, residual confounding by unknown or unmeasured factors may exist. For example, information about gut microbiota composition and dietary habits that could affect TMAO concentrations was not available. The generalizability of the findings is also limited to younger adults and individuals with clinical features other than ACS. Another limitation is the use of single measure of metabolites at baseline that may have limited the precision of their long-term exposure due to the within-individual variability over time (i.e., TMAO). Finally, although the associations between dimethylglycine and MACE risk were internally validated, external replication would give greater validity to the findings.

### Conclusions

This prospective cohort study of ACS patients suggests that high plasma TMAO concentrations are related to the risk of developing MACE and this relationship is substantially mediated by renal function. Our results also extend previous research findings, showing that elevated dimethylglycine concentrations are associated with increased risk of MACE, and that renal function partially mediates this relationship. Understanding the potential interplay of TMAO and dimethylglycine with renal function, and incident MACE, may contribute to novel prevention and treatment efforts in ACS.

## Data availability statement

The original contributions presented in the study are included in the article/Supplementary material, further inquiries can be directed to the corresponding author.

## Ethics statement

The studies involving human participants were reviewed and approved by Joan XXIII University Hospital. The patients/participants provided their written informed consent to participate in this study.

## Author contributions

CP and AB designed the research and were the coordinators of the study. AB recruited patients and conducted the research. CP conducted the statistical analyses. RS-G and CP drafted the paper. All authors revised the manuscript for important intellectual content, read, and approved the final version.

## Funding

CP was recipient of the Instituto de Salud Carlos III Miguel Servet fellowship (Grant: CP 19/00189).

## Conflict of interest

The authors declare that the research was conducted in the absence of any commercial or financial relationships that could be construed as a potential conflict of interest.

## Publisher's note

All claims expressed in this article are solely those of the authors and do not necessarily represent those of their affiliated organizations, or those of the publisher, the editors and the reviewers. Any product that may be evaluated in this article, or claim that may be made by its manufacturer, is not guaranteed or endorsed by the publisher.
