# Supplementary material for: Plasma trimethylamine-N-oxide, its precursors and risk of cardiovascular events in patients with acute coronary syndrome: Mediating effects of renal function
Source: Front Cardiovasc Med. 2022 Sep 21;9:1000815. doi: 10.3389/fcvm.2022.1000815 (PMC9532606; doi:10.3389/fcvm.2022.1000815)

Supplementary Material

**Supplementary Table S1.** List of metabolites identified in the LC-MS/MS method.

| Metabolite | HMDB_ID | Retention time (min) | Precursor ion (m/z) | Product ion (m/z) |
| --- | --- | --- | --- | --- |
| TMAO | HMDB0000925 | 3.54 | 76.08  76.08 | 58.1  42.0 |
| TMA | HMDB00906 | 1.85 | 174.01  174.01 | 118  58.1 |
| Choline | HMDB0000097 | 3.46 | 104.12  104.12 | 60.1  45.1 |
| Betaine | HMDB0000043 | 4.36 | 118.09  118.09 | 59.1  58.1 |
| Dimethylglycine | HMDB0000092 | 3.46 | 104.1  104.1 | 42.1  58.1 |
| L-carnitine | HMDB0000062 | 5.11 | 162.1  162.1 | 60.2  103.1 |

Abbreviations: TMAO, trimethylamine N-oxide; TMA, trimethylamine.

**Supplementary Table S2.** Associations of eGFR with the risk of MACE^1^.

| **MV models** | **HR per increment in eGFR levels** | **P value** |
| --- | --- | --- |
| MV1 | 0.99 (0.98-0.99) | **0.002** |
| MV2 | 0.99 (0.97-0.99) | **0.030** |
| MV3 | 0.99 (0.98-0.99) | **0.003** |
| MV4 | 0.99 (0.98-0.99) | **0.008** |
| MV5 | 0.99 (0.98-0.99) | **0.002** |
| MV6 | 0.99 (0.98-1.00) | 0.163 |
| MV7 | 0.99 (0.98-0.99) | **0.002** |

^1^Values are HR (hazard ratios) with 95% confidence intervals. MV1 adjusted for age, sex, body mass index (kg/m^2^), smoking, hypertension, dyslipidemia, type 2 diabetes, unstable angina, acute ST-segment elevation myocardial infarction, non-ST-segment elevation acute myocardial infarction, statin medication, beta-blockers, oral antidiabetic medication, insulin medication, diuretics, aspirin.

MV2 adjusted for the variables in MV1 plus trimethylamine N-oxide. MV3 adjusted for the variables in MV1 plus trimethylamine. MV4 adjusted for the variables in MV1 plus choline. MV5 adjusted for the variables in MV1 plus betaine. MV6 adjusted for the variables in MV1 plus dimethylglycine. MV7 adjusted for the variables in MV1 plus L-carnitine.

MV, multivariable; MACE, major adverse cardiovascular events. Bold text indicates statistically significant P values.

**Supplementary Table S3.** Internal validation using a bootstrap resampling approach (1000 iterations) of the associations

of baseline individual metabolites concentrations with the risk of MACE^1^.

|  |  | **Tertiles of plasma metabolite concentrations** |  |  |  |  | |  |  |
| --- | --- | --- | --- | --- | --- | --- | --- | --- | --- |
| **Metabolite** | T1 | T2 | T3 | P trend | HR per 1 SD increment | | P value | | |
| **TMAO** |  |  |  |  |  | |  | | |
| Concentrations (µmol/L) | <8.01 | 8.01-<14.96 | >14.96 |  |  | |  | | |
| Cases | 29 | 45 | 57 |  |  | |  | | |
| MV | Ref. | 1.43 (0.80-2.57) | 1.66 (0.92-3.01) | 0.175 | 1.15 (0.92-1.44) | | 0.217 | | |
| **TMA** |  |  |  |  |  | |  | | |
| Concentrations (nmol/L) | <36.09 | 36.09-<70.24 | >70.24 |  |  | |  | | |
| Cases | 38 | 45 | 48 |  |  | |  | | |
| MV | Ref. | 1.13 (0.66-1.96) | 1.10 (0.65-1.88) | 0.813 | 1.01 (0.83-1.23) | | 0.906 | | |
| **Choline** |  |  |  |  |  | |  | | |
| Concentrations (µmol/L) | <10.39 | 10.39-<13.35 | >13.35 |  |  | |  | | |
| Cases | 38 | 42 | 51 |  |  | |  | | |
| MV | Ref. | 0.99 (0.57-1.73) | 0.87 (0.50-1.50) | 0.571 | 1.02 (0.81-1.28) | | 0.864 | | |
| **Betaine** |  |  |  |  |  | |  | | |
| Concentrations (µmol/L) | <35.27 | 35.27-<47.02 | >47.02 |  |  | |  | | |
| Cases | 48 | 38 | 45 |  |  | |  | | |
| MV | Ref. | 0.64 (0.36-1.11) | 0.80 (0.46-1.40) | 0.569 | 0.96 (0.75-1.23) | | 0.770 | | |
| **Dimethylglycine** |  |  |  |  |  | |  | | |
| Concentrations (µmol/L) | <2.69 | 2.69-<3.80 | >3.80 |  |  | |  | | |
| Cases | 31 | 46 | 54 |  |  | |  | | |
| MV | Ref. | 1.53 (0.86-2.75) | 2.16 (1.13-4.16) | 0.028 | 1.41 (1.08-1.84) | | **0.012** | | |
| **L-carnitine** |  |  |  |  |  | |  | | |
| Concentrations (µmol/L) | <42.51 | 42.51-<53.74 | >53.74 |  |  | |  | | |
| Cases | 43 | 39 | 49 |  |  | |  | | |
| MV | Ref. | 0.77 (0.45-1.31) | 1.21 (0.70-2.10) | 0.407 | 1.06 (0.85-1.33) | | 0.592 | | |

^1^Values are HR (hazard ratios) with 95% confidence intervals. A natural logarithmic transformation was applied to the raw values of individual metabolites. Cox regression analysis was used. MV adjusted for

age, sex, body mass index (kg/m^2^), smoking, hypertension, dyslipidemia, type 2 diabetes, glomerular filtration rate, unstable angina, acute ST-segment elevation myocardial infarction,

non-ST-segment elevation acute myocardial infarction, statin medication, beta-blockers, oral antidiabetic medication, insulin medication, diuretics, aspirin. Abbreviations: MV,

multivariable; Ref, reference; MACE, major adverse cardiovascular events; TMAO, trimethylamine N-oxide; TMA, trimethylamine. Bold text indicates statistically significant

P values.

**Supplementary Figure S1.** Spearman’s correlation coefficients between plasma concentrations of trimethylamine N-oxide (TMAO), its precursors and estimated glomerular filtration rate (eGFR).

A

**
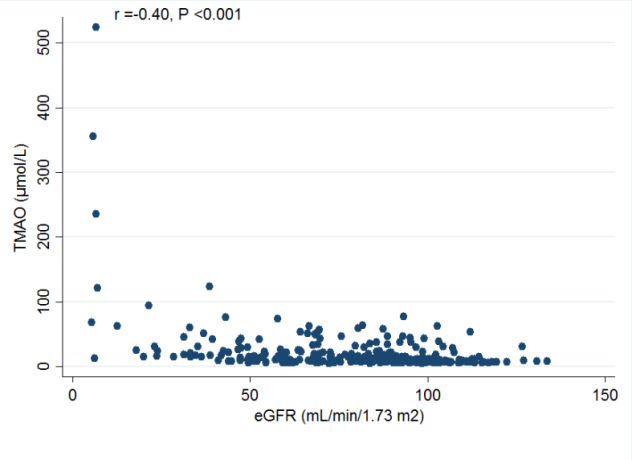
**

B


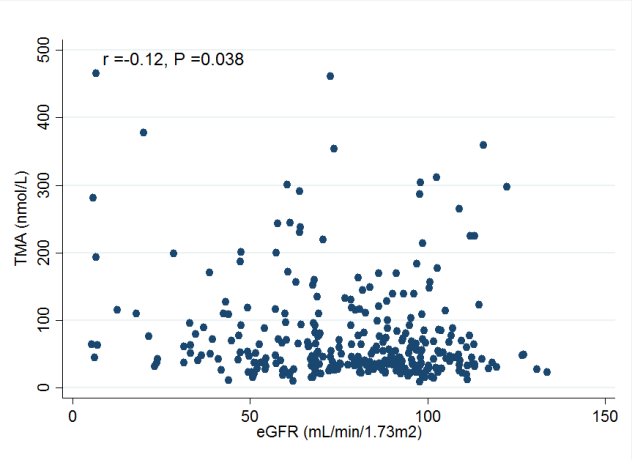


C


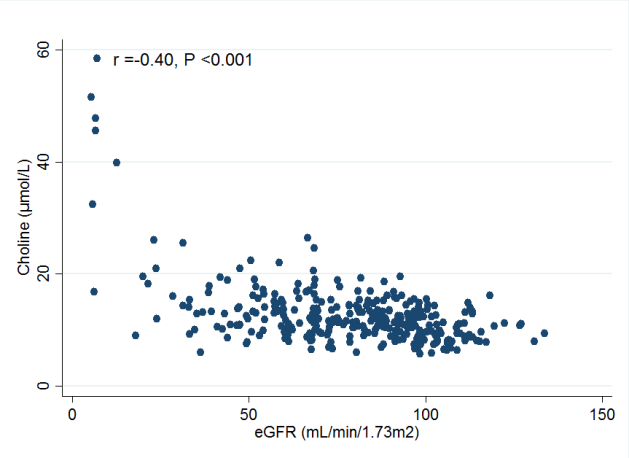


D


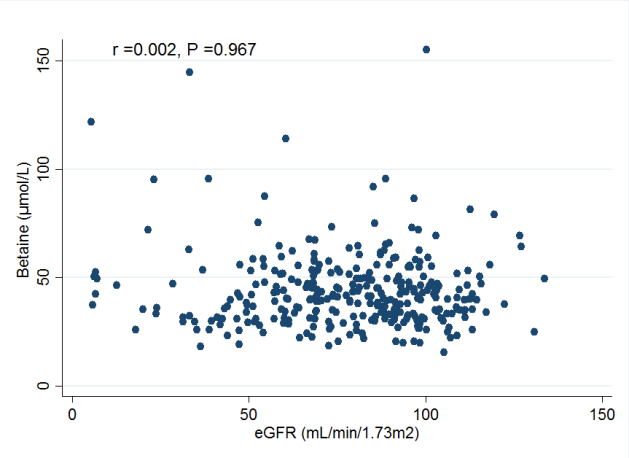


E

**
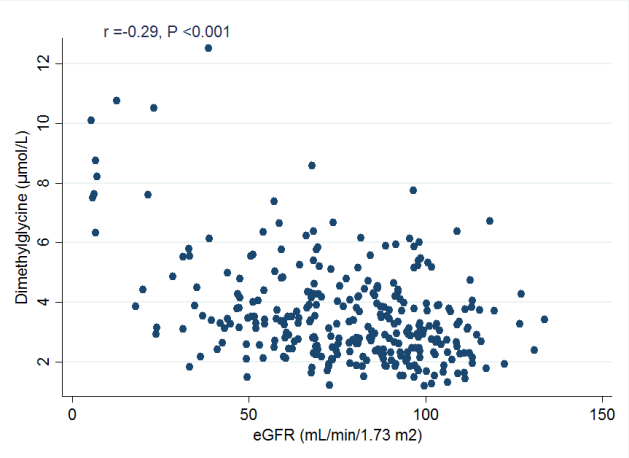
**

F

**
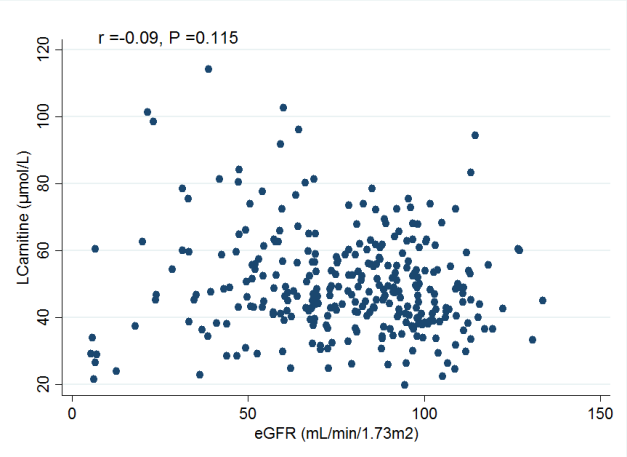
**

**Supplementary Figure S2.** Kaplan-Meier curves of incident major adverse cardiovascular events (MACE) according to tertiles of dimethylglycine, P < 0.001 by log-rank test.


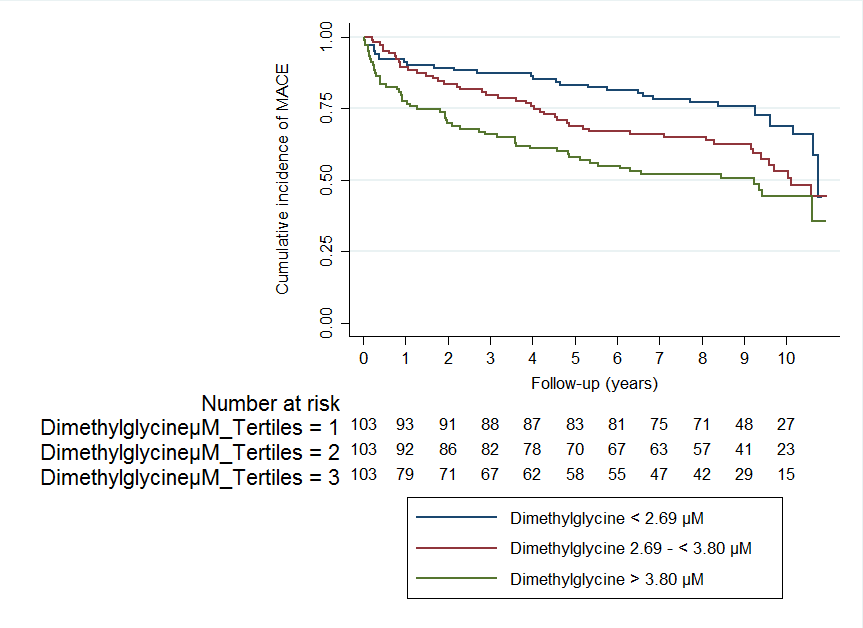


**Supplementary Figure S3.** Spline of concentrations of dimethylglycine and incident major adverse cardiovascular events. Dotted lines are 95% confidence intervals of the spline; horizon line is the reference.


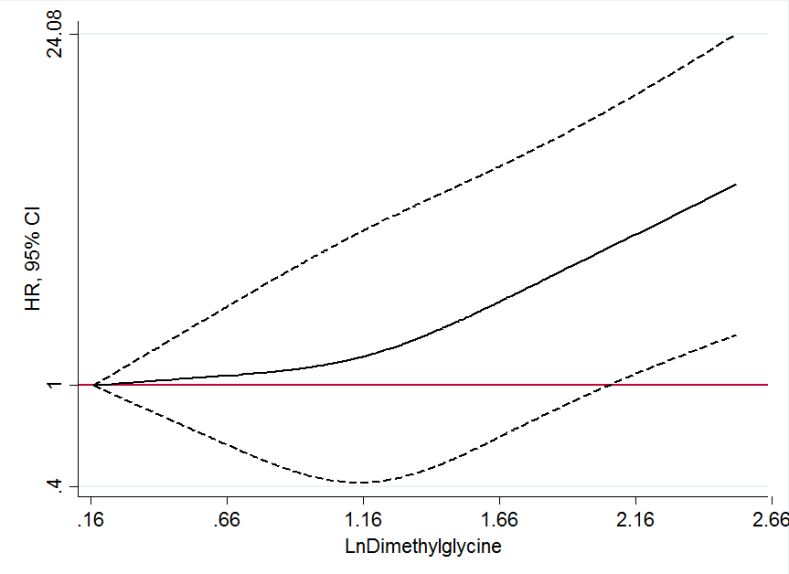

Supplement: Supplementary file 1 [file Data_Sheet_1.docx]
